# Plugging Teeth with Gold

**Published:** 1863-11

**Authors:** B. Wood


### PLUGGING TEETH WITH GOLD.

BY B. WOOD, M. D.

[Read before the Brooklyn Dental Association.]

*Gentlemen of the Association:*—Plugging Teeth with Gold is the subject assigned me for this paper. I would have preferred some other, because unable to see how I was to offer anything new upon this, or more than recapitulate what has already been said, and much better said than I can hope to do. To dress up old things in attractive garb is not my province, content, if able to give expression to facts in a manner to be clearly apprehended.

But although unable by novelty, either of matter or manner, to claim your attention, there is this encouragement that a subject interests in proportion to our knowledge of it; and if the subject interests, the style of treating it is not so material.

Two methods of discussing this subject present for choice, equivalent to two topics in the same language: 1st, *Plugging* Teeth with Gold; 2d, Plugging Teeth with *Gold.* I select the latter.

During the twenty years of my professional practice, I have, by precept and example, advocated plugging teeth with *gold*, and, until the past two years, with gold exclusively.

As to plugging roots and building out teeth with it, I have had little experience. I seldom undertook the treatment and filling of diseased teeth; because, until recently, I had not confidence in the modes of cure proposed or practiced within my observation, and because I had a way of reckoning the public good by the *number* of teeth positively saved. If, therefore, I could surely save half a dozen or more in the time demanded by *one*, diseased and almost worthless, with no surety as to the final result, it seemed better that it should go by the board than to neglect the claims of the former. But our friend, Dr. Atkinson, has taught me the possibility of cure, and therefore the value of treatment — taught theoretically and experimentally — by striking at the *root* of the difficulty, to its bottom, outside and in, figuratively speaking and literally. To him I refer for elucidation of the philosophy and mode of execution.

I confine myself to the advantage or disadvantage of plugging teeth with gold in contradistinction to other materials. But since, when efficiently employed, its preferability to most others is conceded for cases where it can be so employed, or too obvious to need argument, and as I know of none that lays claim to rivalry except amalgam; which, considering the vast amount of work being done with it, and its praises by dentists of high repute, is become truly a formidable rival, we will, for brevity, confine the comparison to this.

If amalgam, in *skillful hands*, can be more securely introduced than gold in skillful hands, and is equally inocuous; if, when *properly* prepared, it uniformly remains sweet and clean; if, rightly compounded and the surplus mercury thoroughly got rid of, and the mass efficiently edulcorated in alcohol, water or other abluent, and the dentinal tubuli be saturated with creosote, it never blackens the teeth, nor oxidizes or tarnishes any more than gold introduced with the requisite care and skill; then it is at least *equal* to gold in utility, and is preferable to it for economy; still more in view of the fact that a far greater number of teeth can be repaired with it in the same time. And, accordingly, those who aspire to doing the greatest good for humanity, ought, in all honesty, to use it and commend it instead of gold. Then plugging teeth with *gold* is of no positive *use*, merely ornamental, and might be left to the jewellers, while the true professional man, earnest in his high vocation, despising mere glitter, confined himself to amalgam.

But *all* this does not accord with my observation of amalgam plugs. For twenty years, I have heard of modes of preparing and introducing them so as not to stain or discolor the teeth; but have yet to find this result verified. I have rarely seen teeth, having amalgam plugs for any considerable time, but that were more or less stained by them. A few exceptional cases there were, and some two or three in which the plugs were not even tarnished. But it did not appear that this was due to any peculiarity in compounding or applying the amalgam, but rather to the condition of the patient's teeth and mouth. So, too, in some cases we find silver plates remaining clean and bright; while, generally, they blacken, and not unfrequently, are soon eroded and destroyed.

It is fair to add, however, that I have not witnessed much of the results of amalgam in New York, during my brief residence in this city, where, being more extensively employed and countenanced by some of the better class of dentists, as well as by others, than in any other place I have ever known, its mode of preparation and introduction has probably been brought to the highest state of perfection practicable. I have seen but one amalgam plug of Dr. Clowes, whose skill in the use of this material, as well as of gold, is acknowledged by all—(a gentleman, by the way, whose intelligence and ingeniousness claim our high admiration)—but the filling, a small one, was of recent date. It was neatly introduced, and had not, as yet, discolored the tooth; but it was quite gray, showing incipient oxidation, and contrasted unfavorably with a tin plug of prior date in an adjoining tooth, which remained clean and bright.

It may be that, by certain adjustment of proportions, the mercury will be held in true and permanent chemical union in the compound, preventing any elimination of it that would discolor the dentine. If so, such proportionment ought to have been found out ere this, and the results should be uniform. But can any say that none of their amalgam fillings ever blacken the teeth?

The volatile nature of mercury, its instability in such combinations, and its weak affinity for the metals associated with it, are shown by the fact that if mixed up into a paste with tin or with *pure* silver, in filings, and the mass be laid aside, in process of time the mercury will be dissipated by evaporation, and the other metals left in loose, dry filings, as at first. So in experimenting with mercury, one's watch and chain may become coated with its vapor, which in time volatilizes off, leaving the surface clean again.

Tests have been made to prove the very small amount of mercury in a plug, by subjecting the paste to pressure and then comparing the weight of the fluid portion expressed with that of the mercury used, etc. If, for instance, ten grains of mercury were used in forming the paste, and eight grains of fluid be squeezed out, it is assumed as proven that but two grains remain in the plug. But the fallacy of this is manifest, since the excess of mercury dissolves a portion of the other metals, which it carries out with it. It is a partial transfer of metals, part of the mercury remaining in the mass, and part of the other metals being carried out with the expressed fluid. The weight of the portion thus expelled may be even greater than that of the mercury used, so that according to this criterion, the quantity left in the plug would be *less* than nothing! Using enough mercury to make the mass fluid at the ordinary temperature, the whole would pass through the strainer. If we combine bismuth with an excess of mercury, the amalgam is fluid; to this we may add a portion of lead, and the result be still fluid, so as to pass through a towel as readily as pure mercury.

On the principle involved in the above, the discoloration of teeth admits of explanation. Mercury, oxidized and finely divided, is black; so are silver and tin. Eliminating itself from the plug, and carrying particles of these metals with it into the dentinal tubuli, it necessarily discolors the tooth. If the plug is imperfect, or contracts owing to a loss of substance, admitting oxygen, the effect is intensified. This is verified by the case of the "cadmium-amalgam" pastes, which shrink more perceptibly and admit oxygen freely. Oxide of cadmium, finely divided, is yellow; as are its salts, precipitated by sulphuretted hydrogen; when, therefore, transported by the mercury into the tubuli, it imparts a yellow stain to the tooth. It is simply a difference of color. In like manner, when decay goes on beneath the plugs, the softened dentine is, in the one case, stained black; in the other, yellow.

If an amalgam of mercury with silver, or with silver and tin, could be so compounded as not to tarnish in the mouth, it would be a phenomenon indeed. Mercury oxidizes and blackens,—so may tin, and silver still more than tin. By what process of preparation is a compound of these metals to be transmuted into a new substance, not liable to tarnish under like conditions?

For these and other reasons, not necessary to enumerate, the advantage of plugging teeth with gold instead of amalgam, is apparent. Doubtless, to fill teeth *well* with amalgam, is better for their preservation than filling them badly with gold; while many can make better plugs with it than they can with gold. And, notwithstanding all the outcry against amalgam, I doubt not that more teeth have been saved by its employment, than would have been done had the same operators resorted to gold. Much of this outcry probably originated in a spirit of persecution, while yet in other cases it was sincere. On the whole, it was beneficial; for it has forced attention to improvement in gold-filling. Had the profession and community rested satisfied with amalgam plugs, where would have been the magnificent results with gold now beheld? And could we succeed in reëstablishing confidence in amalgam, how soon would this higher skill languish for want of stimulus and patronage!

There is another recommendation in favor of gold filling, which ought to have some weight with professional men. Gold, a neat, clean metal, is ready prepared to the operator's hand, and is manipulated before the eyes of his patients. His skill with it is all exercised upon living organs, and is truly surgical. He is during the time at the chair—his proper place. There is no covert or one-side operation about it. In the case of amalgam, his chief time is occupied over the material—compounding, mixing, triturating, squeezing, and washing—to remove something obnoxious. If he works it in his palm before his patient, the ink-spots left excite distrust and aversion. It soils whatever it touches. He himself feels half-ashamed, and must needs perform this part of his work one-side, slyly. Then he mixes and pummels, triturates in a mortar, washes with water, alcohol, etc., expresses the surplus mercury through napkins, towels and chamois, with hand or vice pressure, wasting much labor and "skill"

upon it which might better be expended upon his patient,—all which may impress with ideas of conjuration and legerdemain, and indeed be very skillful in its way; but it is certainly no legitimate part of plugging teeth. It is not surgical or operative.

In this respect, amalgam is the meanest thing we have, except oxichloride of zinc; and, for my part, I always felt mean when dabbling with either! Tin foil we can work with at the chair before our patient; so can we with Hill's Stopping; so also with the Plastic Metallic Filling. The material is prepared to our hand, the operation open to inspection, and all our skill, discretion and delicacy of manipulation exercised upon our patient.

I will mention yet one consideration in favor of plugging with gold (as also some other materials) in preference to amalgam, which is of moment if we look to the advancement of our profession. It requires a greater degree of skill; and that which calls this quality into exercise, perfects it and contributes to our advancement and ennoblement. If we would rise to the highest excellence, we must boldly grapple with what is at first no easy task, nor suffer it to dampen our resolute efforts until the mastery be achieved. The profession will never make progress upon any system of Dentistry-Made-Easy. If there were two substances in other respects equal, the one requiring no skill, and the other its highest exercise, it would be the part of wisdom to employ and commend the latter, if we regard the perfectibility of ourselves and our profession. A becoming spirit of emulation, stimulating all to equal the best in difficult achievements, would make our profession mount upward on eagle wings. Especially ought the younger members of the profession to be incited to exercise themselves upon whatever calls into play their highest faculties. When the difficult has thus become easy to them, they will safely dally with that which is inherently easy.

For this reason, I regard it a dangerous heresy to commend amalgam filling to young practitioners. If encouraged to resort to it in difficult cases, such cases will be apt to become more frequent, until they imperceptibly glide into the exclusive use of amalgam; and from this it is easy to glide, upon the same plane, into the most slovenly use of it.

